# Physical basis of specificity and delayed binding of a subtype selective sodium channel inhibitor

**DOI:** 10.1038/s41598-018-19850-9

**Published:** 2018-01-22

**Authors:** Ben Corry

**Affiliations:** Research School of Biology, Australian National University, Canberra, ACT 2601 Australia

## Abstract

Nerve and muscle signalling is controlled by voltage-gated sodium (Nav) channels which are the targets of local anesthetics, anti-epileptics and anti-arrythmics. Current medications do not selectively target specific types of Nav found in the body, but compounds that do so have the potential to be breakthrough treatments for chronic pain, epilepsy and other neuronal disorders. We use long computer simulations totaling more than 26 *μ*s to show how a promising lead compound can target one Nav implicated in pain perception and specific channels found in bacteria, and accurately predict the affinity of the compound to different channel types. Most importantly, we provide two explanations for the slow kinetics of this class of compound that limits their therapeutic utility. Firstly, the negative charge on the compound is essential for high affinity binding but is also responsible for energetic barriers that slow binding. Secondly, the compound has to undergo a conformational reorientation during the binding process. This knowledge aids the design of compounds affecting specific eukaryotic and bacterial channels and suggests routes for future drug development.

## Introduction

Voltage gated sodium channels (Navs) initiate action potentials in excitable cells by opening in response to small depolarising signals to allow for rapid influx of Na^+^ into the cell. A large number of sodium channel-related diseases including forms of epilepsy^1^, heart disease^2,3^ and enhanced pain syndromes^4,5^, originate from mutations altering the gating or inactivation of these channels that allow elongated action potentials or rapid re-firing of nerve impulses. Thus, sodium channels are the target of many drugs, such as local anaesthetics, that work by blocking sodium channels to stop the influx of Na^+^, thereby preventing the nerve impulse from arising^6^. Similarly, temporary block by anti-epileptic or anti-arrhythmic drugs after firing can prevent further chaotic impulses that occur in seizures and cardiac arrhythmias. There are nine types of Nav in humans which are expressed in different tissues. Nav1.1, 1.2, 1.3 and 1.6 are the main types found in the central nervous system; Nav 1.7, 1.8 and 1.9 are found in the peripheral nervous system; while Nav1.4 and 1.5 are primarily found in skeletal muscle and heart respectively^7,8^. Current local anaesthetic drugs target all human subtypes, but the development of highly selective channel blockers would allow for a range of new clinical applications and a decrease in side effects. For example, loss of function mutations in Nav1.7 are implicated in congenital insensitivity to pain^9^ while gain of function is linked to enhanced pain syndromes^10–12^. Thus, drugs that can selectively block Nav1.7 have the potential to treat chronic and neuropathic pain^13,14^, conditions affecting up to 10% of the worldwide population. Significant progress has been made in gaining subtype specificity^15–20^, though this has not yet translated into clinical success.

Eukaryotic voltage gated sodium channels are comprised of a long protein chain that folds into four homologous domains that surround the central ion conducting pore. Each of these domains contains an independent voltage sensing module made from 4 transmembrane helices (S1–S4) and a pore domain (helices S5–S6) that combines with those in the other domains to form the ion conducting pore. All existing small molecule channel blocking drugs bind within the central pore, in a region commonly referred to as the local anaesthetic site^21–24^. While the publication of a number of structures of voltage gated sodium channels from bacteria^25–34^ and subsequent simulation studies^35–39^ have greatly assisted in defining the nature of this binding site, the extremely high degree of sequence conservation in this region makes it challenging to engineer compounds that display subtype selective binding at this position.

**Figure 1:**
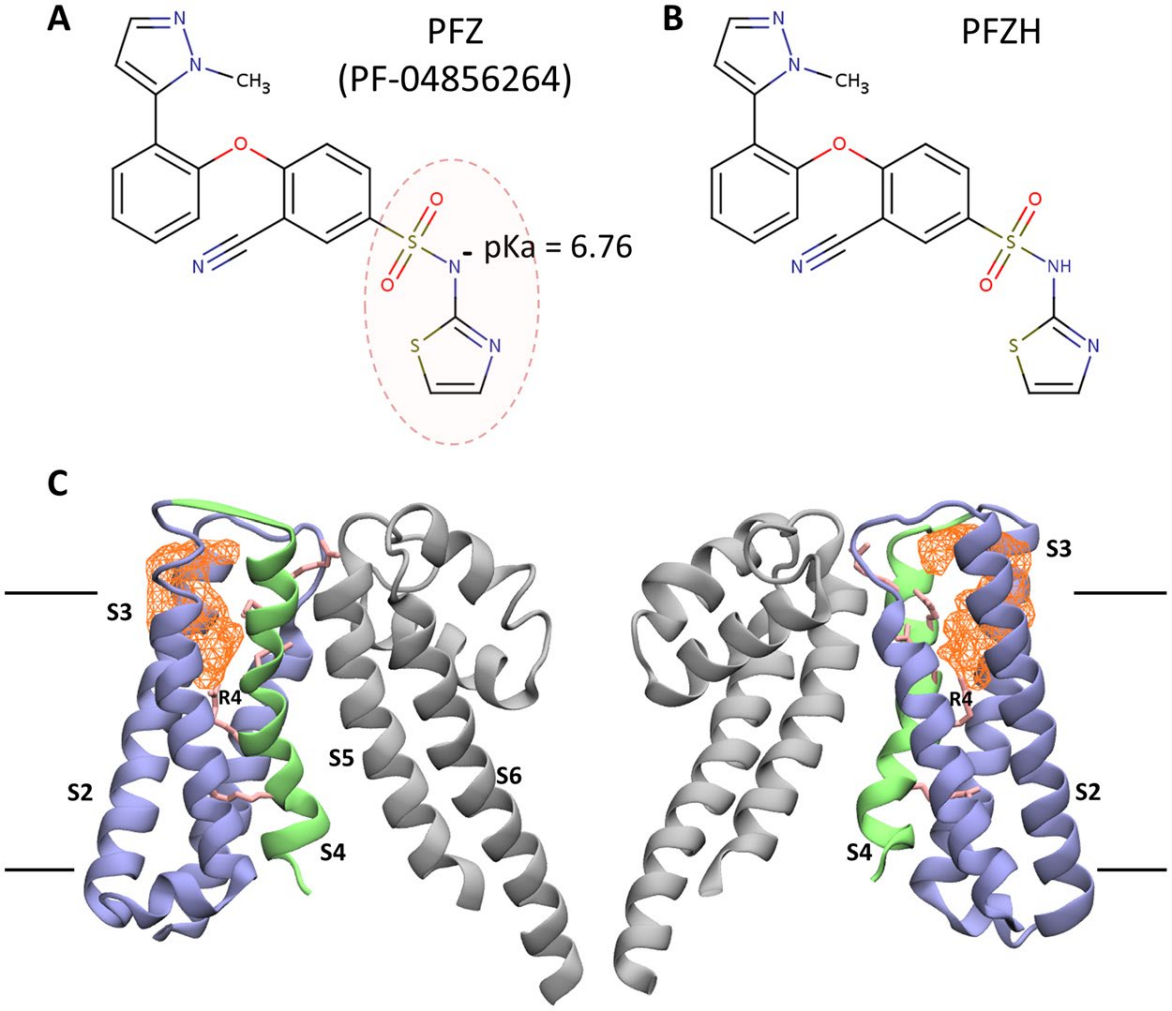
(**A**,**B**) Compounds used in this study. The ‘charged warhead’ of PFZ is circled in red. (**C**) Overall structure of the Nav proteins. The pore domain is shown in grey, the voltage sensor in blue and green and arginine residues in the S4 helix in pink. The water accessible binding pocket is indicated by orange mesh. The approximate location of the lipid bilayer is indicated by the horizontal lines on each side. Only two out of four protein subunits are shown. The structure of the Nav1.7/NavAb chimera is shown in which the voltage sensors (blue/green) from Nav1.7 is spliced with the pore domain (grey) of NavAb.

The aryl sulfanomide compounds PF-04856264 (here called PFZ)^20^, (Fig. 1) PF-05089771 and PF-05198007^40,41^ have recently been described as subtype selective sodium channel inhibitors, having low nm IC50 for Nav1.7 and greater than ten-fold weaker affinity for all other Navs. One end of these molecules carries a negative charge at neutral pH and has been referred to as the ‘charged warhead’. In contrast, the other end of the compounds are hydrophobic in nature, allowing for a range of interactions to form with the protein. PFZ and analogues have been shown by mutation studies to bind to the extracellular side of the voltage sensor^20,40^, an area with greater sequence diversity than the central pore, making this an attractive lead for developing subtype selective channel modulating compounds. More recently the location of binding of a PFZ analogue to a chimeric sodium channel in which a eukaryotic voltage sensing domain was attached to a bacterial pore domain (Nav1.7/NavAb chimera) was characterised using X-ray crystallography^34^. This showed a deeply buried binding site, in which the charged warhead interacts with arginines on the S4 helix of the voltage sensor, commonly known as the gating charges.

While PFZ and analogues have a good binding affinity for Nav1.7, they are also characterised by a very slow on rate. The compound and its derivatives are only active against the inactivated states of the channel^41^, and to measure inhibition the channel is held in the inactivated state for many seconds or very high concentrations are applied^20,34,40^. Direct measurements of the time course of inhibition show rates in the order of hundreds of seconds at 100 nM and limited use dependent inhibition during repeated channel firing^41^ Such long timescales for inhibition will influence the therapeutic utility and dosage of these inhibitors as the channel is only expected to remain in the inactivated state for a few ms in the physiological setting^42^. Thus, gaining an understanding of why it takes so long for the compounds to inhibit the channel and whether this can be overcome is essential to expand the therapeutic range of the compounds.

Here we use more than 26 *μ*s of MD simulations with the channel structures from NavAb^25^ and Nav1.7/NavAb Chimera^34^ to address two questions central to the development of subtype selective channel modulating compounds. Firstly, how is it that binding of PFZ is subtype selective in both eukaryotic and bacterial sodium channels? Secondly, why does it take so long for the compound to reach the binding site and inhibit channel currents?

## Results and Discussion

### Understanding subtype selective binding

To better characterise the binding site of PFZ, we conduct simulations of the compound in both NavAb and the Nav1.7/NavAb chimera, starting with PFZ in the crystallographic binding position. As the compounds bind to the inactivated state of the channel in which the voltage sensors are in an activated (‘upward’) position^20,34^, the existing crystal structures containing activated voltage sensors are ideal starting points for this study.

**Figure 2:**
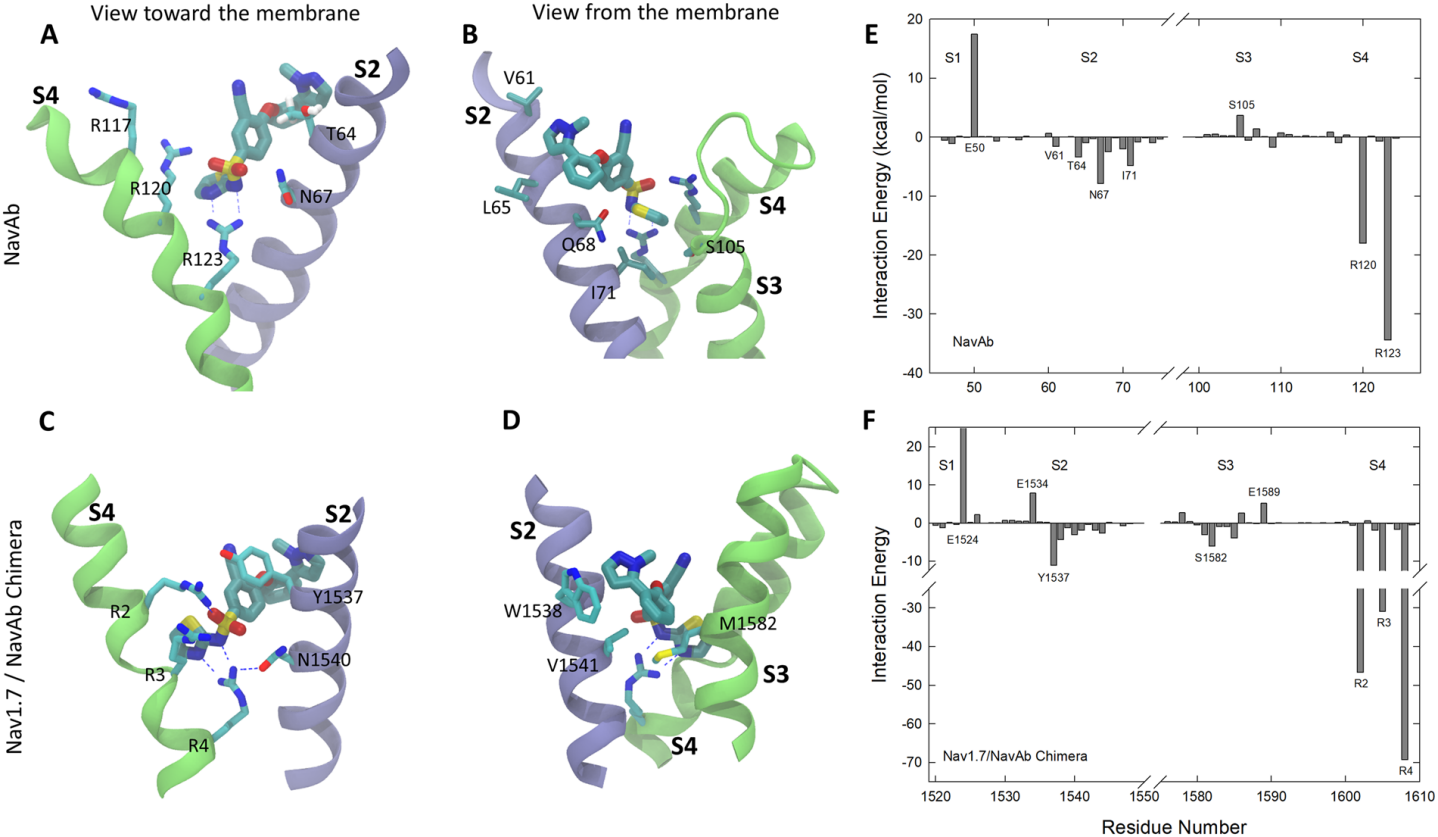
Characterising the crystallographic PFZ binding site in NavAb (**A**,**B**) and the Nav1.7/NavAb Chimera (**C**,**D**). Figures show representative snapshots of each compound in 200–500 ns MD simulations starting with the drug aligned to the crystallographic position as viewed looking from the pore toward the membrane (**A**,**C**) and from the membrane toward the pore (**B**,**D**). Protein residues involved in important interactions with PFZ are indicated and the S2 helix is shown in blue and S3, S4 in green. PFZ-protein interaction energies averaged across the simulations are shown for (**E**) NavAb and (**F**) Nav1.7/NavAb Chimera. Residues with strong interactions are identified.

The high affinity binding site seen for an analogue of PFZ in the Nav1.7/NavAb crystal structure is found to be present in our simulations for PFZ in both NavAb and the Nav1.7/NavAb chimera. PFZ binds strongly and stably to both proteins, with a copy of the compound remaining very close to the starting coordinates in each voltage sensor. As will be shown later, the binding affinity is predicted to be much greater in the Nav1.7/NavAb chimera (*n*M) than in NavAb (*μ*M). In the binding position, PFZ interacts with the gating charge R4, as well as other gating charge residues, as shown in Fig. 2A–D, consistent with the reduction in binding affinity found for PFZ analogues upon mutation of many of the gating charges^34^. In addition, PFZ forms a number of long lasting interactions with other amino acid side chains. As suggested previously, it is likely that PFZ and its analogues block channel currents by trapping the voltage sensor in activated position by directly interacting with the S4 helix. This prevents the protein from returning to the resting state as required for the channel to open in response to further membrane depolarisations.

To highlight the interactions of the compound with the protein, we determine the average interaction energy of the compound with each of the protein residues as shown in Fig. 2E and F. These interaction (potential) energy values should not be used to directly decompose the binding free energy of the compound, but can be used to identify the residues with which the compound directly interacts. This analysis indicates that a number of residues on the S2 and S3 helices take part in binding PFZ, albeit with smaller interaction strengths than the gating charges. In NavAb, these are mostly hydrophobic interactions between the back of PFZ to S2 (eg with T64, I71) along with an attractive interaction between N67 and the charged warhead. In contrast, Nav1.7 is able to form more specific aromatic staking interactions between Y1537 on S2 and the B-ring of PFZ, a residue previously highlighted as critical to generating subtype selective binding^20,34^. In addition there are a number of hydrophobic interactions between residues on S2 and S3 with the hydrophobic end of PFZ (eg with W1538 and M1582).

**Figure 3:**
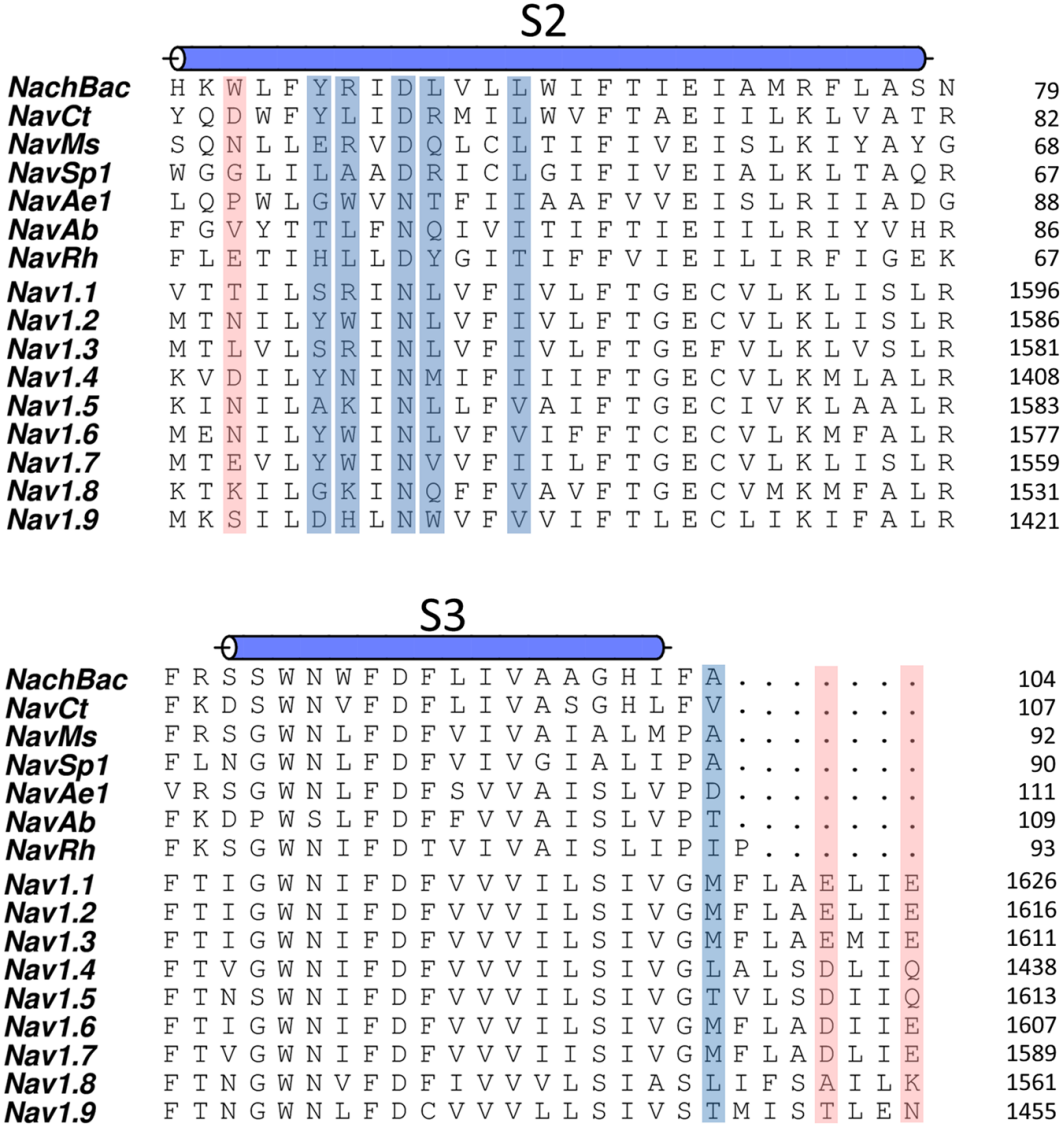
Sequence alignment of bacterial and eukaryotic S2 and S3 helices. Residues involved in strong attractive interactions with PFZ are highlighted in blue, while acidic residue that repel PFZ are indicated in red.

To better understand how the specific interactions between PFZ and the protein can lead to subtype-selectivity, we show a sequence alignment between the prokaryotic and eukaryotic sodium channels in Fig. 3. Highlighting the residues identified as having strong interactions with PFZ (coloured bars) allows for determination of the reasons for specificity for certain channel subtypes. Interactions with the gating charges appear most important for the binding of PFZ, but these are conserved across the sodium channel families. As noted above, one of the next most important interactions of PFZ with Nav1.7 is with Y1537 on the S2 helix. Aromatic residues are also found at this position in Nav1.2, Nav1.4 and Nav1.6 and so are likely to form similar interactions. In contrast, the presence of polar or small hydrophobic residues at this position in Nav1.1, 1.3 and 1.5 may partly explain weaker binding in those cases, as PFZ cannot have favourable stacking interactions with these residues. The favourable hydrophobic interaction of W1538 seen in Nav1.7 can also be expected in a number of subtypes which have the same residue at this position. However, differences may be expected in Nav1.1, 1.3, 1.5 and 1,8 which have basic residues at this location. While N1540 forms a strong interaction with the warhead of PFZ in Nav1.7, this residue is conserved across the human channel subtypes and is therefore unlikely to assist in generating subtype selectivity. Sequence differences in the human subtypes S3 may also aid in yielding subtype selectivity, with the most important interaction found in Fig. 2F being with M1582. Other subtypes have a threonine or leucine at this position that might also form hydrophobic contacts, but of differing strength.

To highlight how sequence differences can yield selectivity, we use MD simulations to predict how mutations at specific positions alter the binding affinity of PFZ. (Table 1) We focus on mutations that convert key residues in Nav1.7 to those found in Nav1.5 as selection between these subtypes has been shown for PFZ^20^. The mutation Y1537A, removing the aromatic stacking interaction seen in Nav1.7, reduces the binding affinity by ~2 kcal/mol. More surprisingly, the mutation M1582T in S3 has an even larger influence on the binding affinity. This shows how hydrophobic interactions at the back side of PFZ can be tuned to generate selective binding. Indeed, single point mutations at this position were previously shown to have a greater influence on binding affinity than the single point mutation Y1537A^34^. Together the sequence difference at these two sites are sufficient to convert the *n*M binding seen in Nav1.7 to *μ*M binding in Nav1.5 as inferred from our free energy calculations (Table 2). As a control, the conservative mutation I1544V is found to have a smaller affect on the binding affinity.

**Table 1:** Change in PFZ binding free energy from protein mutations determined from free energy perturbation calculations.

Protein	Mutation	Change in binding free energy (kcal/mol)
Nav1.7	Y1537A	1.8 ± 0.2
Nav1.7	I1544V	0.8 ± 0.1
Nav1.7	M1582T	2.7 ± 0.3

**Table 2:** Binding affinity of PFZ and analogues to different channel proteins determined from experiment and predicted by the umbrella sampling simulations described here.

Protein	Binding affinity	
	Experimental	Computational
Nav1.7	0.1–100 nM^20,34,40^	96 nM (4–980)
Nav1.5	>10 *μ*M, 25 *μ*M^20,40^	~52 *μ*M*
NavAb	>30 nM^34^	4.8 *m*M (2.2–66)

*Note that the computational prediction for Nav1.5 is based on the relative free energy of a Nav1.7 triple mutant obtained from free energy perturbation, so may not represent the affinity seen for the complete Nav1.5 sequence. Confidence range in computational predictions is determined by integrating the maximum and minimum values of the PMF.

Other residues have previously been implicated as important in dictating the selectivity of binding. W1538, for example, forms strong interactions with PFZ and its mutation can influence the binding affinity^34^. This position is occupied by a basic residue in many channel subtypes and so is likely to have very different interactions with the ‘C-ring’ and tail of PFZ. We do not directly study this mutation here due to the difficulty in converging the simulation results for non charge conserving mutations. The acidic residue at the position of D1586 in Nav1.7 has also been shown to be important for determining the selectivity of compounds for Nav1.3 or Nav1.7^20^. The aspartate at this position sits near to the C-ring of PFZ. While the overall negative change on this residue repels the PFZ warhead, the relatively short side chain prevents direct contact with PFZ. The presence of a longer side chain in the glutamate residue found at this position in Nav1.1, 1.2 and 1.3 would generate a closer contact with the bound compound and require more specific interactions to be formed to allow for high affinity binding.

Although the PFZ binding site is also present in NavAb due to similar interactions with the gating charges, this protein lacks the aromatic interaction with the B-ring (ie Y1537 in Nav1.7) and the attractive hydrophobic interactions on S3 (see Fig. 3). For this reason the binding affinity of PFZ predicted for NavAb is much lower than for Nav1.7 and similar or lower than for Nav1.5 (Table 2). NavAb and Nav1.7 both have an asparagine residue in the binding site that interacts with the PFZ warhead and R123 (N67 in NavAb, N1540 in Nav1.7) which has previously been shown to be important in contributing to high affinity binding^34^. However, in some bacterial isoforms there is an aspartate residue in this position which is likely to limit the binding affinity of PFZ as it will repel the like charged PFZ warhead. To test this hypothesis, we ran equilibrium simulation of PFZ in the binding site of a NavAb N67D mutant. After equilibrating in the binding site mutation converts the attractive interaction of PFZ with N67 (−8 kcal/mol) to a repulsive one with D67 (+26 kcal/mol) as seen in Fig. S1. Furthermore, during the simulations D67 is seen to compete with PFZ for interactions with R123 as shown in Fig. S2. As a result, PFZ is seen to partly move out of the binding site in 2 of the 4 subunits during the 100 ns simulation period, indicative of much weaker binding. It can be inferred from this that PFZ and its charged analogues are unlikely to bind as well to NachBac, NavMs and other aspartate containing proteins as they do to NavAb.

### Explaining the slow rate of binding

While simulations of PFZ in the binding site help to explain the origins of subtype specificity, they cannot directly determine why it takes PFZ so long to inhibit the channel. To gain a first appreciation of the time taken for the compound to find the binding site we conduct long *μ*s equilibrium simulations in the presence of a high concentration of PFZ (so called ‘flooding’ simulations). Despite there being a large number of copies of the compound and four potential binding sites (one in each voltage sensor), none reach the binding site within the time frame of these simulations. This is consistent with the slow on rate seen experimentally, as typically the channel needs to be held in the inactivated state for many seconds prior to measuring inhibition. PFZ is, however, rapidly attracted to many spots on NavAb (Fig. S4), with mM affinity sites at the extracellular and intracellular side of the voltage sensor and in a hydrophobic pocket behind the intracellular end of the S6 helices. (Fig. S5). These sites could act as transition sites en-route to the position seen crystallographically or could have a functional influence (especially if the compound can act intracellularly). Our simulations also show that PFZ likes to partition into the bilayer from the aqueous phase with the charged warhead pointing out toward the aqueous phase. The free energy at the edges of the simulation system plotted in Figure S4 indicates that there is a weak free energy minima is present around the lipid headgroups (−2 kcal/mol relative to the aqueous phase). This is consistent with the compound preferring to partition into the bilayer rather than remaining in the aqueous phase.

**Figure 4:**
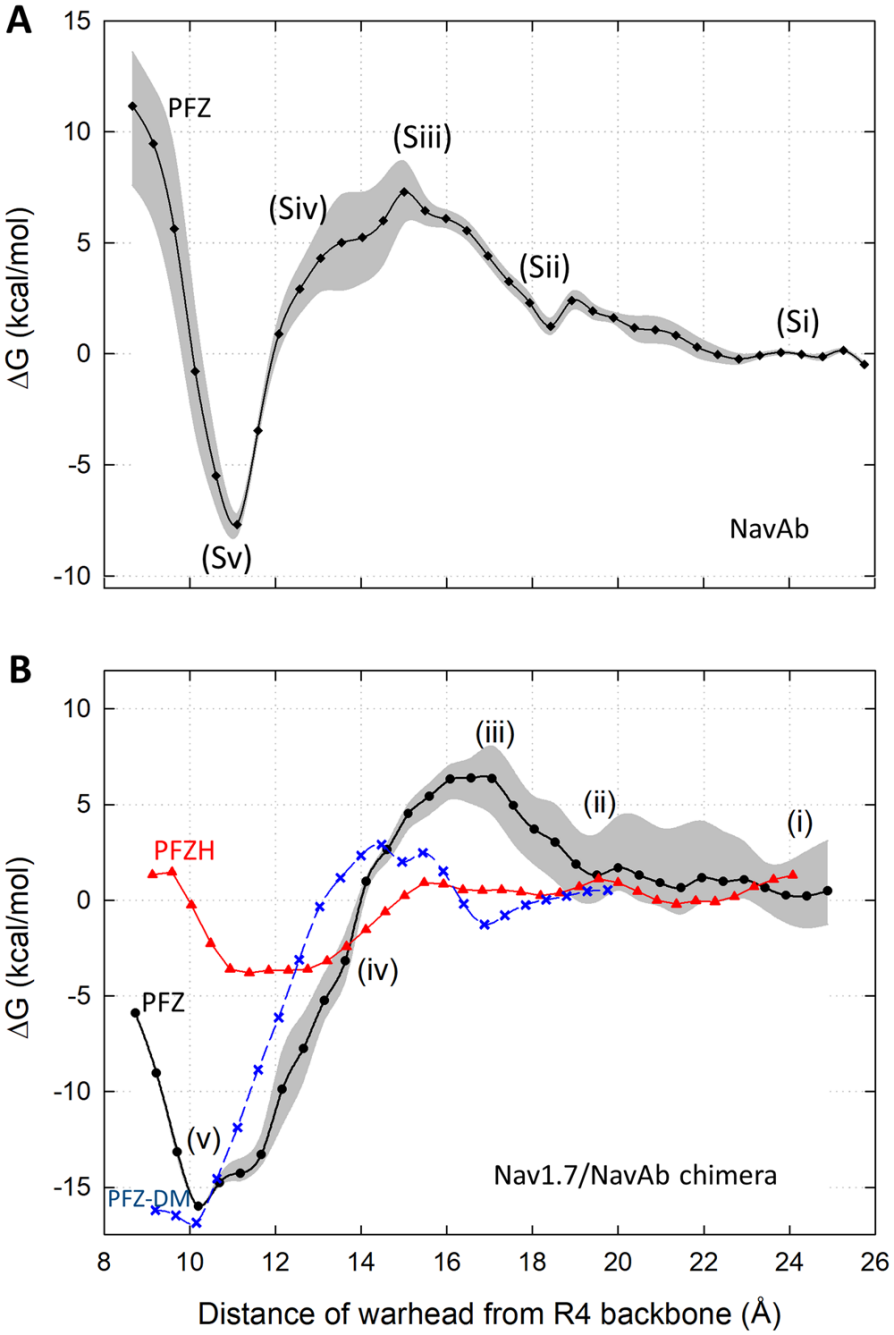
Free energy (PMF) for PFZ to reach the crystallographic binding site in (**A**) NavAb and (**B**) the Nav1.7/NavAb chimera as derived from umbrella sampling simulations. The uncertainty is shown by the grey area on each plot. Locations of the snapshots shown in Figs 5 and S6 are indicated. Also shown is the PMF for the neutral form of the compound (PFZH) in the Nav1.7/NavAb chimera (red triangles line) and the PMF for the D1586A E1589Q double mutant (blue symbols and line).

As binding takes longer than the time frame we can reach in equilibrium simulations, we use the approach of umbrella sampling to determine the location and size of the energetic barriers that slow access to the binding site. We first construct a free energy for the binding process as a function of the distance of PFZ from the binding site. As can be seen in Fig. 4, PFZ faces large energetic barriers in the order of 7 kcal/mol to reach the binding site in both NavAb and the NavAB/Nav1.7 chimera, indicative of time constants of binding in the order of 10 s of *μ*s. The peak of the barrier arises when PFZ is about 6 Å away from the binding site. The umbrella sampling results also allow for the binding affinity of PFZ to each protein to be approximately determined. Binding is much stronger in the Nav1.7/NavAb chimera (~100 nM) than in NavAb (5 mM). The predicted binding affinity in Nav1.7 is in the same range as previous measurements for PFZ (28 ± 5 nM)^20^ and not as strong as for an analogue (0.1 nm)^34^ as indicated in Table 1.

**Figure 5:**
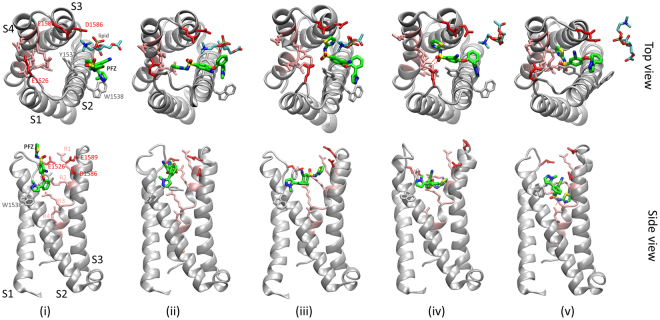
Steps involved in reaching the binding site in the NavAb/Nav1.7 Chimera. Each step corresponds to the position indicated on the PMF in Fig. 4. (**i**) The hydrophobic portions of the compound interact with hydrophobic residues on the outer face of S2: Y1537 W1538 V1541 (also M1582 and F1583 on S3), while the polar warhead points toward the aqueous phase. (**ii**,**iii**) To move toward the binding site, PFZ must displace lipid in the voltage sensor cleft (lipid headgroup shown) and move past the acidic residues D1586 and E1589. The positive choline head of the lipid assist with this by chaperoning passage of the negative warhead past the negative residues. Additional factors making this step difficult are that the compound looses its attractive hydrophobic interactions on S2 and the top of the warhead has to reorient such that the oxygen atoms rather than the terminal ring point toward the gating charges. (**iv**) The negative portions of the warhead directly contact R2 and R3, stabilising it inside the protein cleft. (**v**) Finally, the compound slides easily into the binding site in constant contact with R2 before locking into interactions with R4. Gating charge residues are shown in pink, key acidic residues in red, key hydrophobic residues in grey. PFZ is coloured by atom type with carbons in green.

The umbrella sampling results indicate that binding of PFZ occurs in a number of steps from which the reasons for the slow on rate can be derived (Figs 5, S6). Firstly the compound must partition in to the lipid, a step that is thermodynamically favourable as seen in the flooding simulations discussed earlier. Then, PFZ must diffuse within the bilayer and associate with hydrophobic residues such as W1538 on the outer face of the S2 helix which forms the outermost portion of the protein in the membrane (step (i) in Fig. 5). This is also energetically favourable as the compound can make stronger interactions with the protein exterior than the lipid. Indeed, both these steps are seen to occur spontaneously in the *μ*s flooding simulations. The negatively charged warhead of the compound then has to swing into the cleft in the voltage sensor, and displace lipid that has previously been shown to interact with the R1 gating charge. In doing this, PFZ has to pass a number of acidic residues (E1526, D1586, E1589 in Nav1.7, or E50, E114 and E172 in NavAb) before it can reorient to contact the positive gating charges in S4. Together, these factors generate the free energy barrier seen in Fig. 4 that slows binding. Once contact is made with the gating charges, the compound can easily slide down to the final binding position due to the strong electrostatic interactions formed with each of the gating charges.

**Figure 6:**
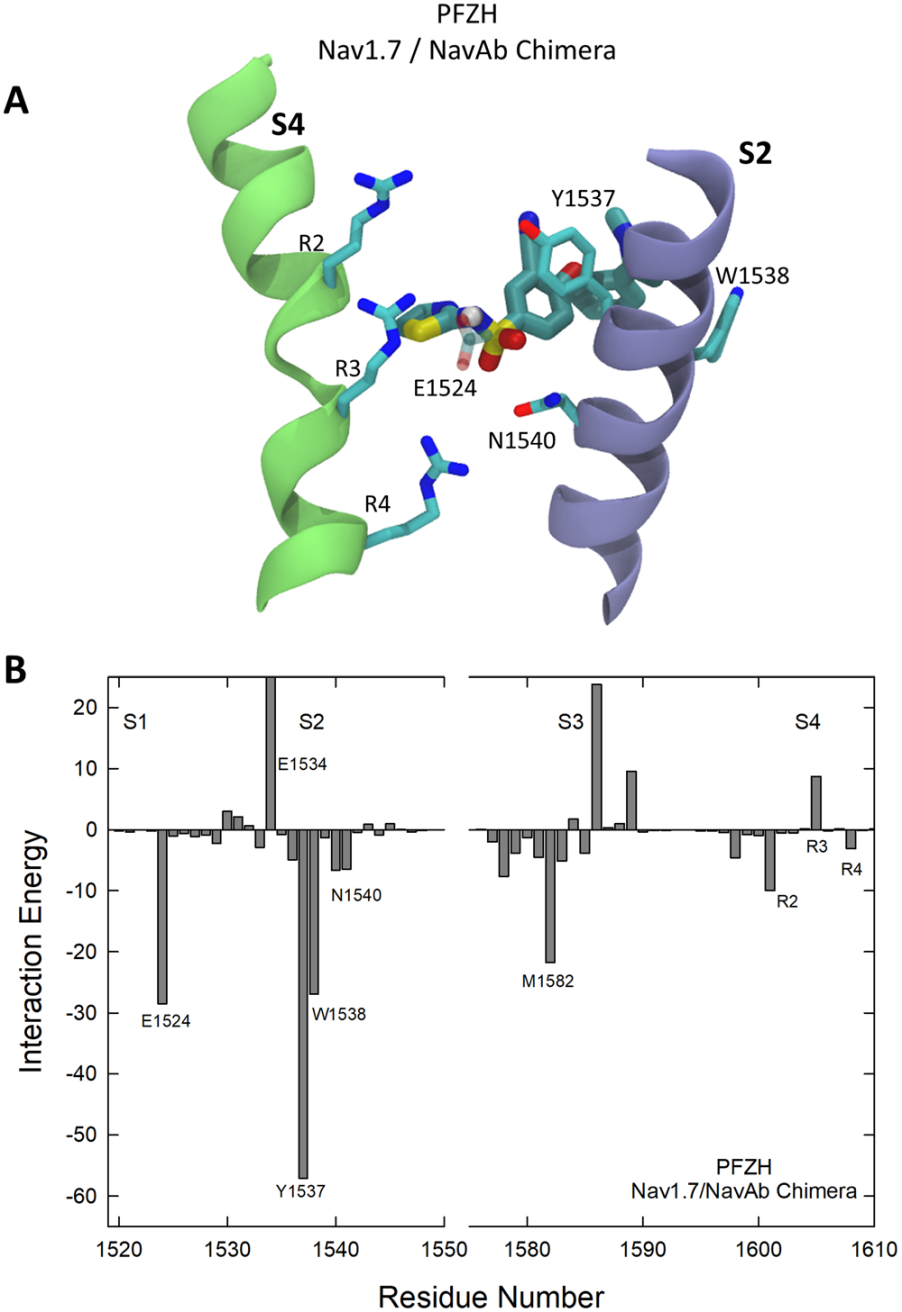
(**A**) Snapshot of the PFZH in the binding site and (**B**) interaction energies of PFZH with nearby protein residues. Residues with strong interactions with PFZH are indicated.

A significant cause of the the energetic barrier seen in the 1D PMF is the electrostatic repulsion of the charged compound with acidic residues on the protein. To prove this fact, we calculate two additional PMFs, one for PFZ in a mutant protein (Nav1.7/NavAb chimera D1586A E1589Q) in which two of the acidic residues are neutralised and one for the neutral form of the compound (PFZH) in the Nav1.7/NavAb. The double mutant has the effect of reducing the free energy barrier in the PMF, which can be expected to speed up the binding rate. As seen from Fig. 4 the neutral compound PFZH also faces only a small energetic barrier (~1 kcal/mol) to reach the binding site. However, this comes at a price, as the binding affinity of this compound is much lower (~2 mM) than for the charged form as it does not interact strongly with the gating charge arginine residues. This is shown explicitly in Fig. 6A in which the position of the neutral compound in the site indicates little interaction with with R4. The plot of interaction energy with nearby protein residues (Fig. 6B) also highlights a much reduced attraction to the gating charges. This in turn allows the compound to move up slightly to have more favourable interactions with Y1537 and W1538, and to form occasional H-bonds with E1524. The reduced binding free energy seen clearly in the PMF makes sense given that the neutral compound will have reduced electrostatic interactions with the protein. Although a neutral compound is an attractive route for speeding binding, unless the strong attraction to the gating charges can be replaced with other specific interactions, the affinity will be greatly reduced.

While our analysis of the 1D PMFs show a consistent pattern, there may be entropic penalties in reorienting the drug that are overlooked in this analysis, as degrees of freedom orthogonal to the chosen reaction coordinate get averaged over. The steps involved in binding indicated in Fig. 5 suggest the importance of reorientation of PFZ en route to the binding site. To better quantify this effect, we plot the mean and standard deviation of the orientation of the molecule as a function of its position in Fig. S8. A dramatic reorientation of the warhead is visible for PFZ in the WT protein, and to a lesser extent, for PFZ in the D1586A E1589Q mutant. No such reorientation takes place for PFZH. Further analysis shows that this reorientation is largely accounted for by a rotation of an internal dihedral joining the phenyl and sulfonyl components of the compound. (Fig. S8D–F). As can be seen in Fig. 5, this reorientation involves a transition from a *cis* to *trans* conformation of the molecule.

**Figure 7:**
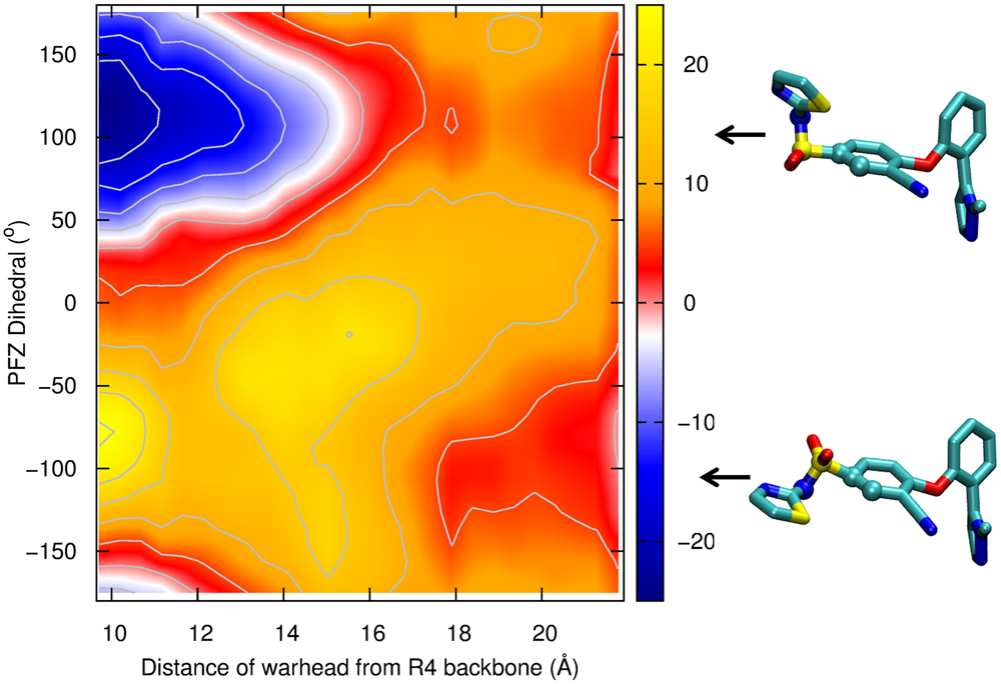
Free energy (PMF) for PFZ to enter the binding site as a function of both the distance of the compound from the site and the internal dihedral angle of the phenyl-sulfonyl bond of PFZ itself. Results are obtained from 2D umbrella sampling simulations. The location of the binding site is shown by the dark blue region at the top left. Snapshots showing the shape of the molecule at minima in the dihedral angle are shown on the right.

To better account for this reorientation, we construct a two dimensional PMF as a function of both the distance of PFZ from the binding site and the dihedral orientation with an additional set of umbrella sampling simulations. As suggested in Fig. 7 and confirmed by a separate simulation of PFZ in bulk (Fig. S9), both the *cis* and *trans* orientations of the molecule are approximately equally favoured in bulk, but there is a significant barrier (5 kcal/mol) for the compound to reorient. On the way into the binding site, however, the *cis* conformation is favoured by more than 5 kcal/mol. In contrast, to reach the binding site, the compound has to adopt the *trans* conformation. This reorientation of the molecule creates an additional 5 kcal/mol barrier to the binding process, which when combined with other factors such as passing the acidic residues yields a total energetic barrier of 13 kcal/mol. The height of this barrier yields a time constant for binding in the range of tens to hundreds of ms. Thus, the combined effect of both the acidic residues and the conformation reorientation may explain the slow rates of binding seen experimentally.

In this study we have addressed two challenges facing the development of subtype-selective sodium channel inhibitors. The first is how to generate highly selective binding. While PFZ harnesses strong interactions with the conserved gating charges to generate high affinity binding, sequence differences at a number of additional key sites can change the binding affinity by many orders of magnitude. Tailoring a small molecule to optimise interactions with these residues in different channel subtypes can yield highly selective binding to specific eukaryotic and prokaryotic channels. The second challenge is to increase the rate of binding of existing voltage sensor inhibitors. We demonstrate two factors that slow the binding of PFZ and its analogues: the need for the negatively charged compound to pass a number of like charged acidic residues and to undergo a conformational change on the way to the binding site. Knowledge of these factors suggests avenues to improve the binding kinetics. Although the charge on PFZ is critical to generate high affinity binding, removing this charge, or utilising zwitterionic molecules may increase the rate of binding. Optimising the interactions of neutral compounds with residues on S2 and S3 may allow for a compromise of moderate affinity binding with more rapid onset. Charged voltage sensor inhibitors may also be more usefully targeted at channels such as Nav1.8 and Nav1.9 that, as shown in Fig. 3, do not include the same acidic residues shown to slow binding in Nav1.7. Alternatively, modifications of the inhibitor structure that restrict its conformational flexibility to make it more likely to adopt the conformation required in the binding site may also increase the binding rate.

## Methods Summary

The NavAb and Nav1.7/NavAb chimera proteins were placed in pre-equilibrated POPC bilayers and solvated in TIP3P water with 250 mM NaCl to create systems of approximate size 72 × 72 × 82 Å. All simulations were run using NAMD with the CHARMM36 force field for proteins and lipids with CMAP correction. Parameters for PFZ and PFZH were developed to be consistent with the CHARMM force field as described in the supplementary material.

The binding sites were characterized using equilibrium simulations starting with 4 copies of PFZ, one in each binding site. Hunting for binding sites was conducted using long simulations with PFZ starting in the aqueous phase. The free energy of drug entry is determined using the method of umbrella sampling with the distance of the negatively charged warhead from the backbone of the R4 gating charge used as the collective variable. Dissociation constants were determined by integrating these curves from bulk to the binding site. The change in the binding free energy of PFZ due to the mutation of specific protein residues is calculated by combining the free energies of multiple alchemical transformations obtained using the method of free energy perturbation.

Full details of the simulations methods are available in the supplementary material.

## Electronic supplementary material

Supplementary Information
