# Supplementary material for: Physical basis of specificity and delayed binding of a subtype selective sodium channel inhibitor
Source: Sci Rep. 2018 Jan 22;8:1356. doi: 10.1038/s41598-018-19850-9 (PMC5778059; doi:10.1038/s41598-018-19850-9)
Supplement: Supplementary file 1 — Supplementary Information [file 41598_2018_19850_MOESM1_ESM.pdf]

# Physical basis of specificity and delayed binding of a subtype selective sodium channel inhibitor

Ben Corry

## Supplementary data

## Methods

### Simulation systems and parameters

A pre-equilibrated and solvated system of closed NavAb in a POPC membrane was obtained from Martin and Corry et al.[1] In this system the coordinates of closed/pre-open NavAb were obtained from the protein databank (pdb accession number 3RVY).[2] A similar simulation system was made for the Nav1.7/NavAb chimera by taking the crystal structure (accession code 5EK0) [3], placing it in a pre-equilibrated POPC membrane and solvating in a TIP3P water and adding 250 mM NaCl to create a system with dimensions of 72 × 72 × 82 Å. The systems were equilibrated as for our previous simulations [1, 4]: the protein was first held fixed while water and lipid were allowed to equilibrate for 2 ns. Then the protein alpha carbons were then restrained by a harmonic potential with force constant reducing from 10 kcal/mol to 0.1 kcal/mol in 4 steps over 10 ns, followed by 100 ns of unrestrained simulation.

The Nav1.7/NavAb chimera structure was obtained with PFZ in the binding site, however, this had to be introduced in the site in NavAb for those simulations starting with the compound bound. In order to allow the protein and compound to equilibrate to one another and avoid problems with steric clashes we initially placed a harmonic constraint on the protein alpha carbons and on all the heavy atoms of PFZ. Simulations were run for 2ns each with the force constants of the harmonic constraint at 10, 5, 2, 1, 0.4, 0.1 kcal/molÅ$^2$ and then with no harmonic potential for 8ns prior to data collection.

All simulations were run using NAMD [5] with constant temperature (310 K) and pressure (1 atm) maintained using Langevin dynamics and a Langevin piston. The particle mesh Ewald method was employed to compute the complete electrostatics of the system.[6] All bonds to hydrogen were kept fixed allowing the use of 2 fs timesteps. Protein and lipid parameters were obtained from the CHARMM27[7] and CHARMM36[8] force fields respectively with CMAP correction. Ion parameters were obtained from Joung and Cheatham.[9] Parameters for PFZ and PFZH were developed to be compatible with the CHARMM CGENFF forcefield. [10] To do this, initial parameters were derived using the automated atom typing and parameter assignment tools.[11, 12] Refinement of the partial charges for atoms with large penalties was done by by conducting ab-initio geometry optimisation and then fitting the charges to the electrostatic potential all using Hartree Fock theory and the 6-31+G* basis set with the program GAUSSIAN03 [13] and the Merz-Kollman (MK) electrostatic fitting method [14, 15].

A summary of all simulations is shown in Table S1.

### Characterising the crystallographic binding site

Four copies of PFZ are aligned with the position seen in Nav1.7/NavAb chimeric structure, one in each of the four voltage sensors. Equilibrium simulations are run for 200 ns for NavAb and 500 ns for Nav1.7/NavAb chimera. The average interaction (potential) energy of PFZ with each protein residue is determined from the last 100 ns of each simulation using the NAMDEnergy plugin of VMD.[16] These interaction energies cannot be used to directly determine or decompose the binding free energies (which is best done from the PMF calculations) but help to identify residues involved in protein-compound binding.

| Simulation | Run length | Notes |
|---|---|---|
| ***Characterising binding site*** | | |
| PFZ in NavAb site | 200 ns | |
| PFZ in Nav1.7 chimera site | 500 ns | |
| PFZ in NavAb N67D site | 100 ns | |
| PFZH in Nav1.7 chimera site | 100 ns | |
| ***Effect of mutations on binding affinity (FEP)*** | | |
| Y1537A | 80 ns × 2 directions × 6 repeats | Total 960ns |
| I1544V | 80 ns × 2 directions × 6 repeats | Total 960ns |
| M1582T | 80 ns × 2 directions × 6 repeats | Total 960ns |
| ***Flooding to see entry and binding*** | | |
| PFZ - NavAb | 1060 ns | 69 mM PFZ conc. |
| ***Energetics of entry - Umbrella sampling*** | | |
| PFZ in NavAb | 17 ns × 35 windows | Total 595 ns |
| PFZ in Nav1.7 chimera | 100 ns × 53 windows | Total 5300 ns |
| PFZH in Nav1.7 chimera | 30 ns × 42 windows | Total 1260 ns |
| PFZ in Nav1.7 chimera D1586A E1589Q | 80 ns × 53 windows | Total 4240 ns |
| PFZ in Nav1.7 chimera 2D PMF | 20 ns × 486 windows | Total 9720 ns |
| PFZ in bulk water | 7ns × 18 windows | Total 126 ns |

Table S1: Summary of the simulations run for this study.

**Hunting for binding sites**

PFZ was placed at high concentration (69 mM) in the aqueous region of the simulation as shown in Fig. S3 and then allowed to freely interact with the protein for 1060 ns (so called 'flooding simulations'). The most commonly occupied positions, specific drug-protein interactions and free energies are characterised. To do this, the simulation system is divided into 2x2x2 Å grid squares and the probability of finding the centre of mass of a PFZ molecule in each box is determined. Free energy plots were derived from this, taking advantage of the four-fold symmetry of the system, using

$$\Delta G = -kT \ln P(x, y, z) \tag{1}$$

Where P(x,y,z) is the normalised probability of finding PFZ in each grid box. two dimensional projections of the data are made by integrating the probability across one dimension between two bounds and recalculating the free energy as above.

**Access to the crystallographic site**

The free energy of drug entry is determined using the method of umbrella sampling.[17] For the one-dimension PMFs, the distance of the negatively charged warhead from the backbone of the R4 gating charge is used as the collective variable. This was defined as the distance of the centre of mass of the 9 heavy atoms of the 'warhead' end of PFZ, to the centre of mass of the R4 alpha carbon and the two alpha carbons each side of this. A harmonic potential with force constant 15 kcal/molÅ$^{-2}$ is used to restrain the distance for a series of windows, spaced 0.5 Å apart in the range 9.5-25 Å for NavAb, and 9.5-30 Å for Nav1.7/NavAb chimera which has a larger VS domain. An additional set of umbrella windows with force constant 25 kcal/molÅ$^{-2}$ was used in the range 11-16 Å for PFZ-Nav1.7/Navab chimera to ensure overlap between positions in neighbouring windows in the region where the forces are changing rapidly. To prevent having to sample all orthogonal coordinates in the bulk region the warhead was restrained in a cylinder of radius 10 Å in the z-direction centred on an axis passing through the centre of mass of the 3 alpha carbons described above. The position data from each window is collectively analysed using the weighted histogram analysis method (WHAM) [18, 19] to obtain the final potential of mean force (PMF) using the implementation of Grossfeld [20]. As each simulation contains 4 PFZ molecules (one in each protein subunit), there is the potential to gain 4 separate PMFs from each set of simulations. However, a complete PMF requires physical overlap between the positions sampled within each umbrella window. Although good overlap is found along the collective variable itself, in some cases the Cartesian coordinates of adjacent windows do

not overlap. PMFs of specific compounds in which there is not overlap in both the CV and the Cartesian coordinates were excluded, leaving 3 complete PMFs for the PFZ-NavAb simulation and one for the PFZ-NavAb/Nav1.7 chimera, 4 for the PFZH-NavAb/Nav1.7 chimera and 1 for the PFZ-NavAb/Nav1.7 D1586A E1589Q mutant.

To determine if the umbrella windows have run for long enough to converge the PMF and to determine the appropriate amount of equilibration time to discard, we recalculate the PMF from different continuous subsets of the simulation data as shown in Figure S7. For the PFZ-Nav1.7/NavAb chimera, excluding more than the first 20 ns of data does not significantly influence the final result, but excluding less than this creates a noticeable effect. Thus, for the final plots we exclude the first 20 ns of each umbrella window as equilibration time. It is also evident that the PMF does not change as we extend the simulations beyond 80 ns suggesting that the final results have converged to within the uncertainties discussed below. A similar analysis showed that shorter simulations allowed for convergence for the other PMFs as indicated in Table S1.

Statistical uncertainty in the data can be computed using Monte-Carlo bootstrap analysis in which the PMF is recalculated for a number of random subsets of the data and the standard uncertainty of this determined. However, this yields negligible uncertainties which are barely visible on the PMF plots and do not represent the real uncertainty that comes from the slow timescale motions of the protein. A better estimate of the uncertainty can be gained by examining the reproducibility of the PMF itself. Thus, for the PFZ-NavAb system, the uncertainty is calculated from the range of values found in the 3 independent PMFs determined for the separate compounds in the simulations as described above. As we only have a single PMF for the PFZ-NavAb/Nav1.7 chimera we instead plot the maximum and minimum values of the PMF at each position found when recalculating the PMF from all possible windows of data $\geq$ 20 ns.

In order to calculate the 2D PMF as a function of distance from the binding site and dihedral angle, we conduct umbrella sampling with an additional bias potential on the phenyl-sulfonyl dihedral angle. Umbrella windows were constructed along the distance variable using a force constant of 20 kcal/molÅ$^{-2}$, spaced 0.5 Åapart in the range 10-23 Å, and while the dihedral windows were spaced 20 degrees apart with a force constant of 0.01kcal/mol rad$^{-2}$. Due to the large number of windows, each was run for 20ns with the first 5ns discarded as equilibration time prior to analysis. The PMF of the PFZ dihedral in bulk water was constructed by first equilibrating the compound in a 30 x 30 x 30 Å water box with a single counter ion. Umbrella windows were spaced 20 degrees apart with a force constant of 0.01kcal/mol rad$^2$. Runs lasted 7 ns per window with the first 2 ns discarded as equilibration prior to analysis.

**Dissociation constants**

The dissociation constant for PFZ in and NavAB/Nav1.7 chimera and in NavAb were determined by integrating the potential of mean force from bulk to the binding site following the method described previously [21, 22]

$$K_d^{-1} = 1000\pi r^2 N_A \int_{z_0}^{z_1} \exp[-w(z)/kT]dz \tag{2}$$

where $K_d$ is the dissociation constant, $r$ is the radius of the constraining cylinder, $N_A$ is Avagadro's number, $w(z)$ is the PMF, $z_0$ and $z_1$ are the values of the collective variable in the bulk region and binding site respectively, $k$ is Boltzman's constant and $T$ is the temperature.

The dissociation constant for PFZ in Nav1.5 was estimated by combining the value found for Nav1.7 with the free energy of the three mutations reported in Table 1 that converts the Nav1.7 binding site to be similar to Nav1.5.

**Binding free energy changes from mutations**

The change in binding free energy of PFZ from mutating specific residues is calculated by combining the free energies of alchemical transformations using the thermodynamic cycle below [23] (Eq. 3).

$$\begin{array}{ccc} protein_{WT} & \xrightarrow{\Delta G^{free}_{pert}} & protein_{mutant} \\ \Delta G^{WT}_{bind} \downarrow & & \downarrow \Delta G^{mutant}_{bind} \\ protein_{WT} : drug & \xrightarrow[\Delta G^{bound}_{pert}]{} & protein_{mutant} : drug \end{array} \quad (3)$$

Using this, the change in binding free energy of PFZ generated by the mutation, $\Delta\Delta G$, can be calculated from two separate simulations in which the side chain of a single amino acid is slowly morphed from one species to another. The free energy change is calculated with with PFZ present ($\Delta G^{bound}_{pert}$) and without PFZ ($\Delta G^{free}_{pert}$) and combined as follows to give the total change in binding free energy caused by the mutation:

$$\Delta\Delta G = \Delta G^{mutant}_{bind} - \Delta G^{WT}_{bind} = \Delta G^{bound}_{pert} - \Delta G^{free}_{pert}. \quad (4)$$

The free energy change of the mutation in the individual simulations is calculated using the method of free energy perturbation (FEP) [24]. To do this the mutation is broken into 40 $\lambda$ windows of equal size run for 2 ns each (including 0.5 ns of equilibration). Each mutation is conducted in both directions and repeated 6 times to ensure reproduceability, yielding a total of 960 ns for each mutation. Uncertainties in the final values are determined from the standard error of the repeated simulations. Due to the strong interaction of PFZ with the gating charges, the compound remains in the binding site in all cases, avoiding some of the complications often seen in this kind of simulation.

**Time scale for binding**

The time constant for binding was caculated using transition state theory using a previously published approach [25, 26]. In this, the rate constant is given by

$$k_{bnd} = \frac{\omega_0}{2\pi} \exp\left(-\frac{\Delta G_b}{kT}\right) \quad (5)$$

where $\Delta G_b$ is the hight of the free energy barrier in the PMF and $\omega_0$ is the frequency of the oscillations of the compound along the direction of the PMF defined as

$$\omega_0 = \sqrt{\frac{\partial^2 PMF(z)/\partial z^2}{m_{eff}}}\Bigg|_{z=z_0} \quad (6)$$

where $z_0$ is the location of the local minimum in the PMF on the outside of the energy barrier and $m_{eff}$ is the effective mass of the compound calculated from its average velocity $\langle v^2 \rangle$ in simulations when at $z_0$ using the equipartition theorem $\mathrm{m}_{eff}\langle v^2 \rangle = kT/2$

## Additional Figures

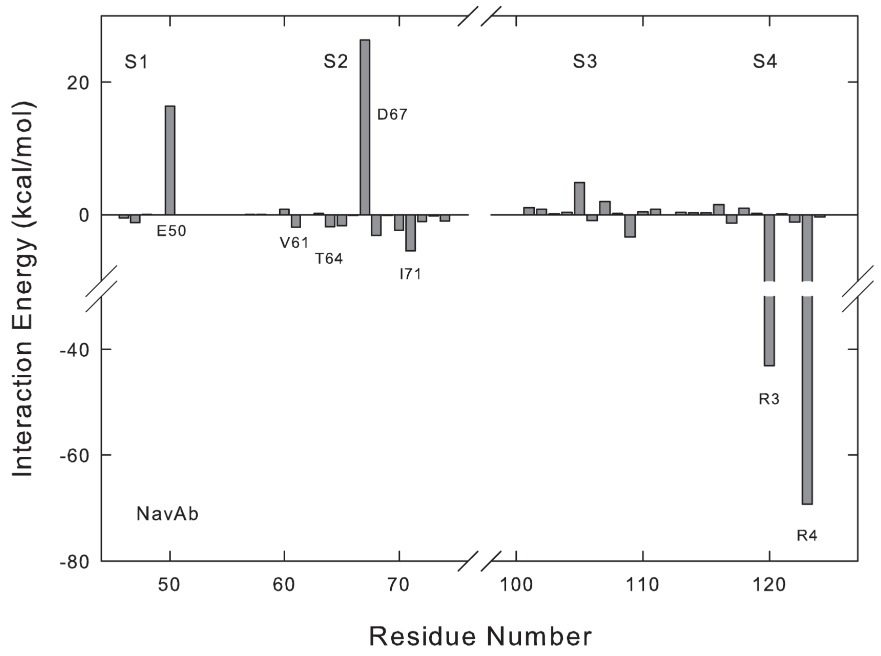


Figure S1: Interaction energies of PFZ with nearby protein residues in the NavAb N67D mutant. Residues with strong interactions with PFZ are indicated.

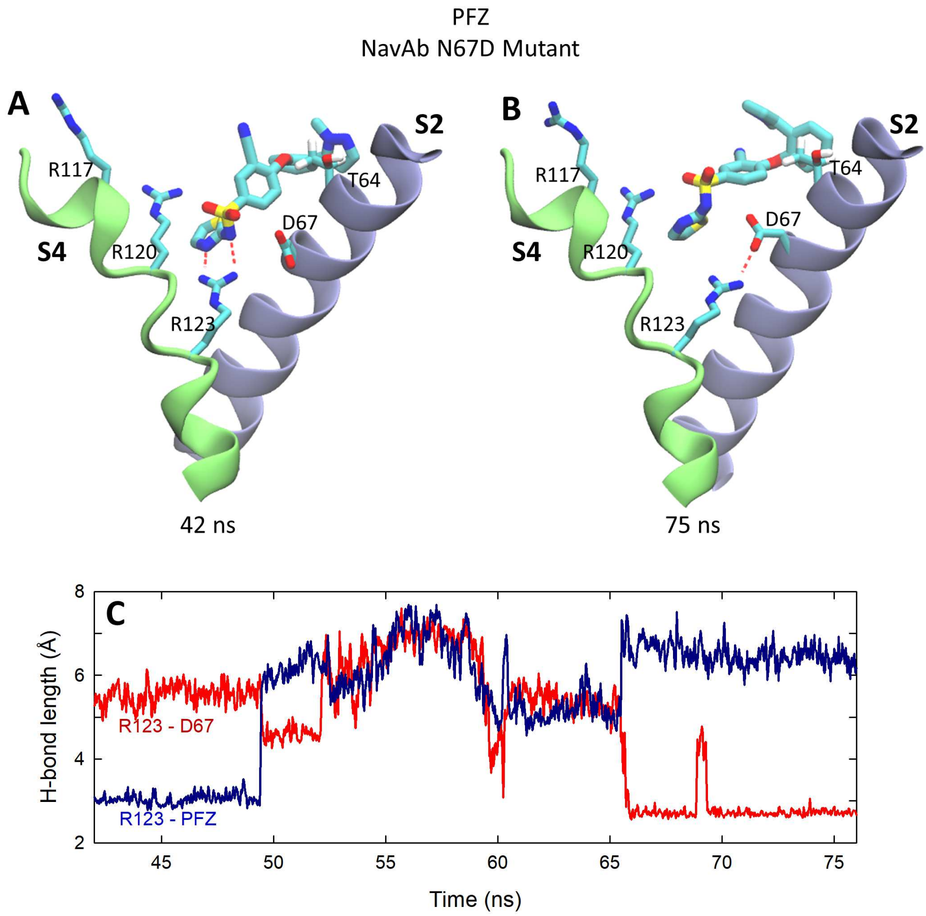


Figure S2: Influence of the N67D mutation on PFZ binding to NavAb. (A) Snapshot of PFZ in the binding site after 42 ns in an equilibrium simulation in which PFZ starts in the binding site as in the WT protein. (B) Snapshot after 75 ns in which D67 is seen to compete with PFZ for interactions with R123. (C) Heavy atom - heavy atom H-bond distances plotted over part of the simulation for (blue) the R123 terminal nitrogen to PFZ warhead nitrogen and (red) R123 terminal nitrogen to D67 terminal oxygen.

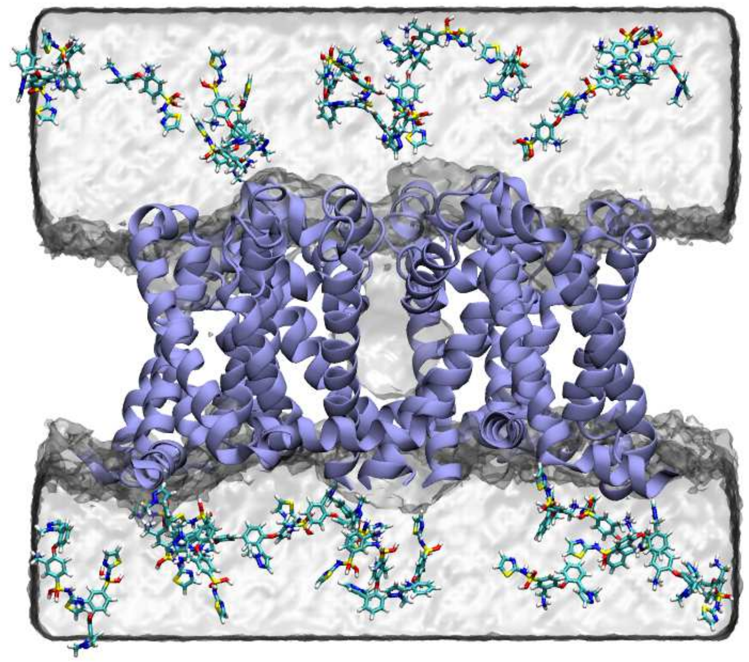

Figure S3: Simulation system used for the flooding simulations. Drugs are initially assigned random coordinates in the aqueous phase as shown at an average concentration of 69 mM. Protein is shown in blue ribbons, the region occupied by water by the transparent surface. Lipid (POPC) and ions (300 mM NaCl) are not shown.

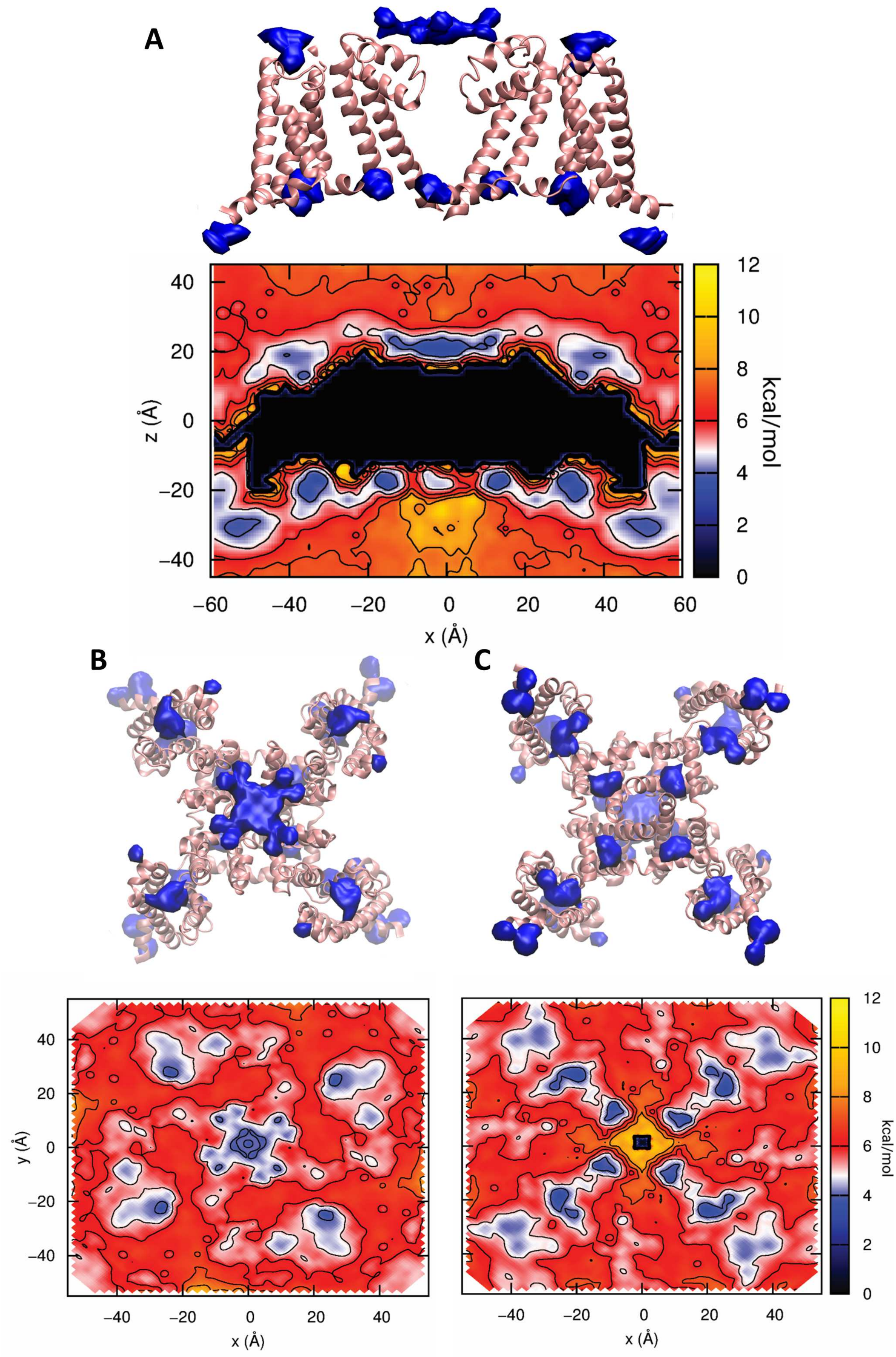

A
B
C
z (Å)
x (Å)
y (Å)
kcal/mol

Figure S4: Hunting for binding sites. Free energy surface for PFZ around NavAb. (A) Side view, Data for $-10 < y < 10$ used only. (B) xy projection using extracellular data ($z > 0$ data only). (C) xy projection using intracellular data ($z < 0$ data only). On the graphs, blue regions represent low free energy areas where the drug is likely to be found. The location of these low free energy regions are shown in the 3D model above each graph. Four main binding regions are seen: the external voltage sensor site, above the selectivity filter, an intracellular site behind S6, and an intracellular site in the voltage sensor. The region above the selectivity filter is broad and reflects a general region of electrostatic attraction and may not be a clearly defined interaction site. The strength of binding in these sites is in the range of 3 kcal/mol (5-10 mM)

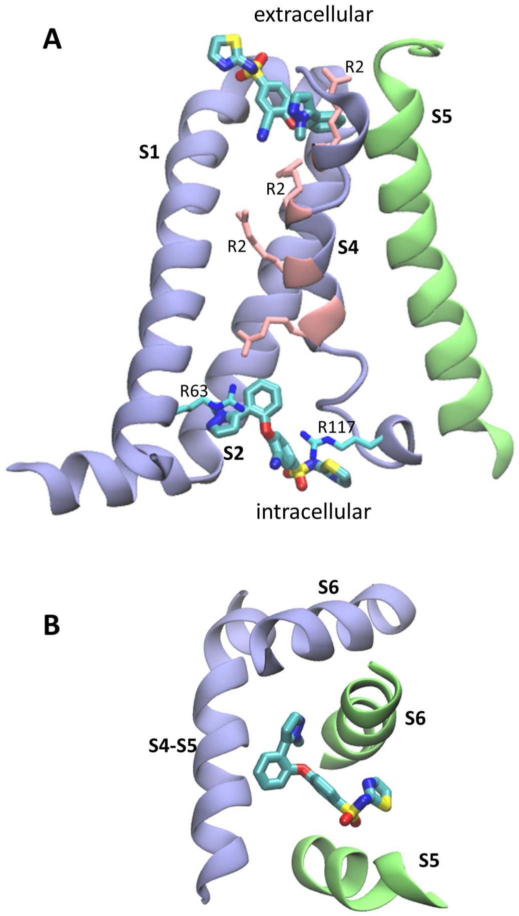


Figure S5: Low affinity binding sites of PFZ in NavAb identified in flooding simulations. (A) Extracellular and intracellular voltage sensor binding sites identified in Fig S4 in the context of the entire voltage sensor, with the basic charged residues on the S4 helix indicated in pink and the S5 helix of the pore domain shown in green. (B) intracellular S6 site shown as viewed from inside the cell.

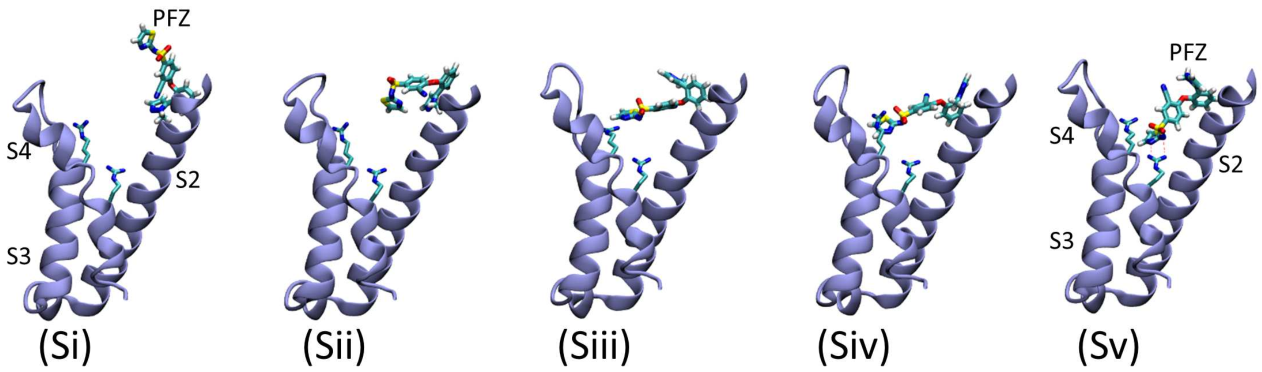


Figure S6: Steps involved in PFZ entering the binding site in NavAb. The compound approaches the binding site by a similar path in all 3 simulated binding events (one in each voltage sensor). (Si) Initially the compound is at the lipid /water interface with the warhead pointing into the aqueous phase. The hydrophobic portions of the compound interact with hydrophobic residues on S2: V61, Y62, T64, L65. (Sii) Next the warhead pivots toward the protein cleft. (Siii) Then the compound has to rotate to change to orientation of the nonpolar end and the warhead has to displace a lipid to interact with R120 yielding the largest energetic barrier. Once the compound can form favourable interactions with R120 (Siv) it can slide deeper into the site to form the final H-bonds to R123 (Sv).

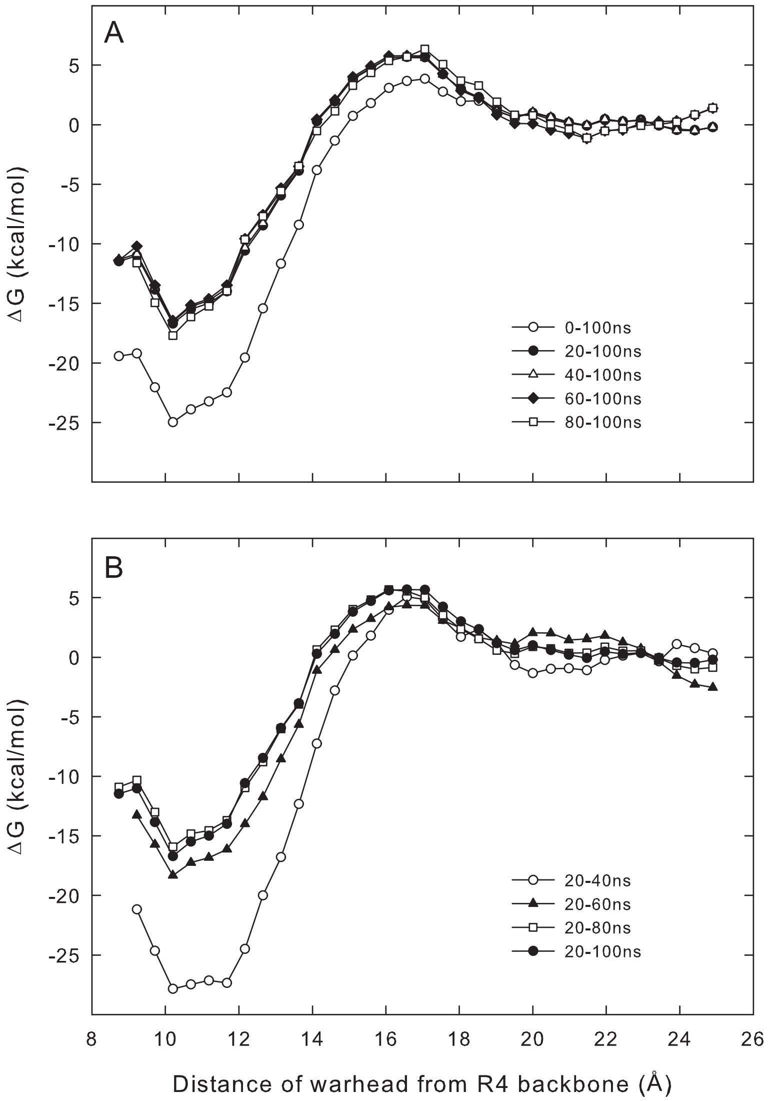


Figure S7: Equilibration and convergence of the PMF results for PFZ in the Nav1.7/NavAb chimera presented in Fig. 4. (A) Results are shown using different lengths of equilibration time prior to collecting data for analysis as indicated in the legend. (B) Results are shown as the length of the simulations is extended from 40 ns to 100 ns as indicated in the legend.

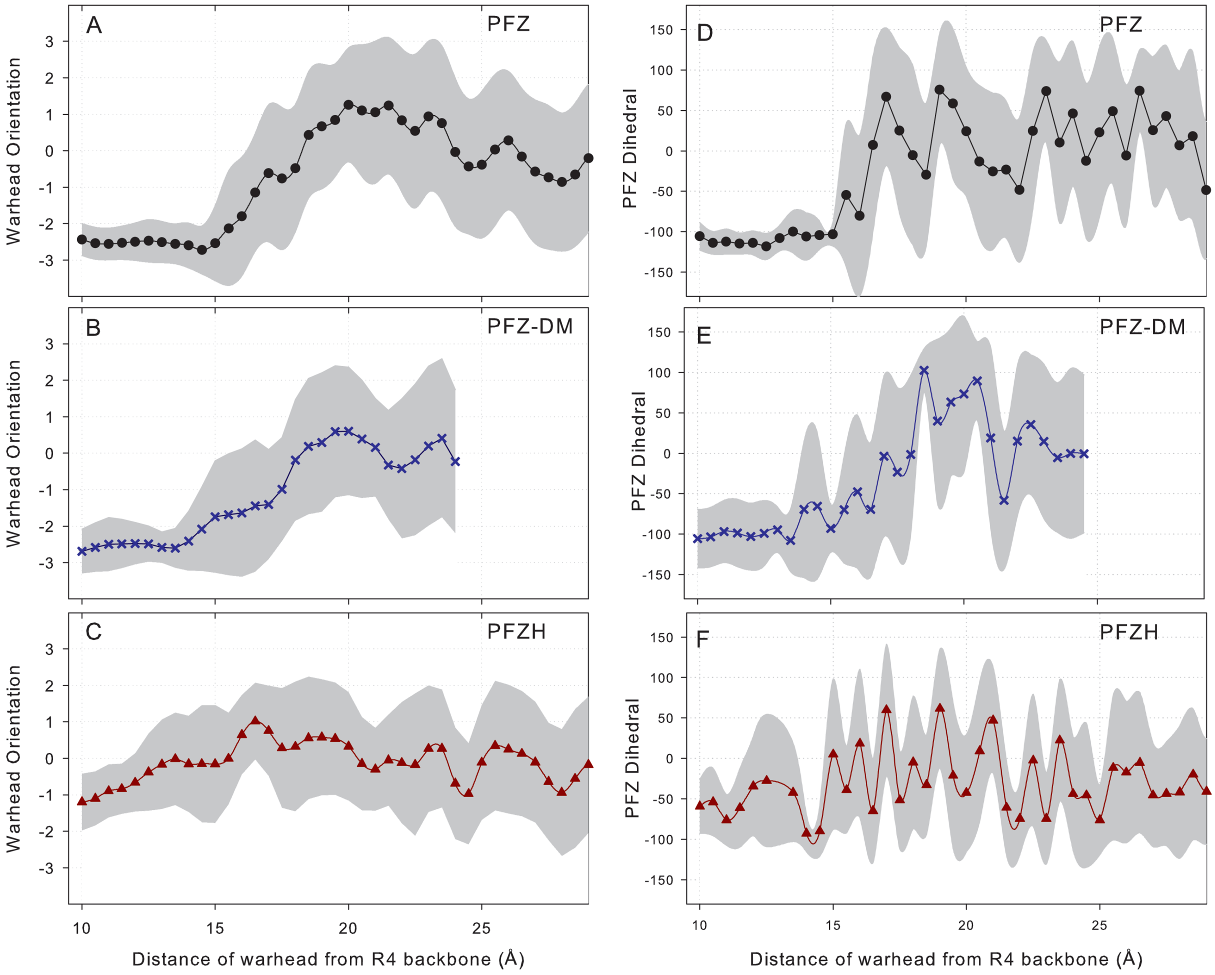


Figure S8: Orientations sampled by PFZ enroute to the Nav1.7 binding site. The orientation of the warhead relative to the protein is shown as a function of the distance from the site for (A) PFZ in the WT protein (B) PFZ in the D1586A E1589Q double mutant, and (C) PFZH in the WT protein. This orientation is defined as the projection of the vector joining the two sulfur atoms in PFZ onto the x axis of the simulation system, after aligning all voltage sensor domains to that of the voltage sensor in chain A. VChanges in the value of this orientation measure, reflect changes in the orientation of PFZ relative to the protein. The values adopted by the sulfonyl-phenyl dihedral is also shown as a function of distance from the binding site for (D) PFZ in the WT protein (E) PFZ in the D1586A E1589Q double mutant, and (F) PFZH in the WT protein. In each case the average value is shown along with the standard deviation of values adopted by the four copies of each compound at each position.

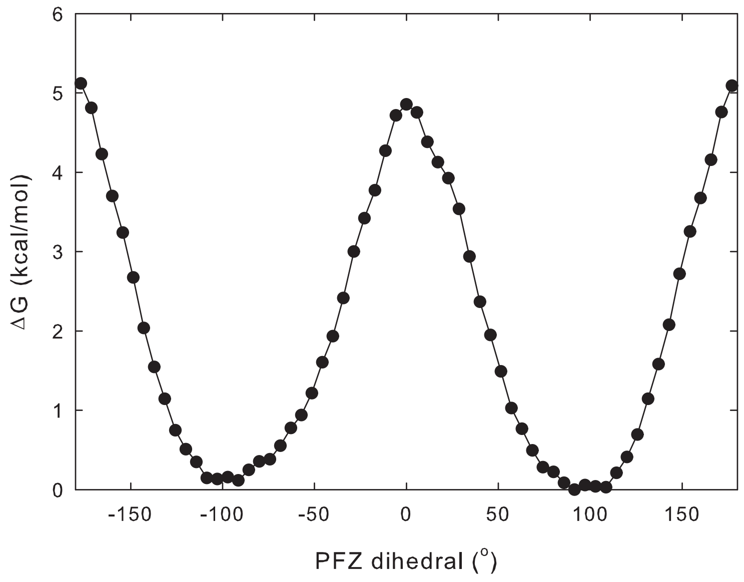


Figure S9: Potential of mean force for the internal phenyl-sulfonyl dihedral of PFZ in bulk water as calculated from umbrella sampling simulations with PFZ in a water box.

# References

[1] LJ Martin, R Chao, and B Corry. Molecular dynamics simulation of the partitioning of benzocaine and phenytoin into a lipid bilayer. *Biophys. Chem.*, 185:98–107, 2014.

[2] J Payandeh, T Scheuer, N Zheng, and W A Catterall. The crystal structure of a voltage-gated sodium channel. *Nature*, 475(1476-4687):353–358, 2011.

[3] S Ahuja, S Mukund, L Deng, K Khakh, E Chang, H Ho, S Shriver, C Young, S Lin, JP Johnson, P Wu, J Li, M Coons, C Tam, B Brillantes, H Sampang, K Mortara, KK Bowman, KR Clark, A Estevez, Z Xie, H Verschoof, M Grimwood, C Dehnhardt, JC Andrez, T Focken, DP Sutherlin, BS Safina, MA Starovasnik, DF Ortwine, Y Franke, CJ Cohen, DH Hackos, CM Koth, and J Payandeh. Structural basis of Nav1.7 inhibition by an isoform-selective small-molecule antagonist. *Science*, 350(6267):aac5464, 2015.

[4] Ben Corry. Na+/Ca2+ selectivity in the bacterial voltage-gated sodium channel NavAb. *PeerJ*, 1:e16, 2013.

[5] J. C. Phillips, R. Braun, W. Wang, J. Gumbart, E. Tajkhorshid, E. Villa, C. Chipot, R. D. Skeel a nd L. Kale, and K. Schulten. Scalable molecular dynamics with NAMD. *J. Comp. Chem.*, 26:1781–1802, 2005.

[6] U. Essmann, L. Perera, M. L. Berkowitz, T. Darden, H. Lee, and L. G. Pedersen. A smooth particle mesh Ewald method. *J. Chem. Phys.*, 103:8577–8593, 1995.

[7] A. D. MacKerell Jr., D. Bashford, M. Bellott, R. L. Dunbrack Jr., J. D. Evanseck, M. J. Field, S . Fischer, J. Gao, H. Guo, S. Ha, D. Joseph-McCarthy, L. Kuchnir, K. Kuczera, F. T. K. Lau, C. Mattos, S. Michnick, T. Ngo, D. T. Nguyen, B. Prodhom, W. E. Reiher III, B. Roux, M. Schlenkrich, J. C. Smith, R . Stote, J. Straub, M. Watanabe, J. Wiorkiewicz-Kuczera, D. Yin, and M. Karplus. All-atom empirical potential for molecular modeling and dynamics studies of proteins. *J. Phys. Chem. B.*, 102:3586–3616, 1998.

[8] J. B. Klauda, R. M. Venable, J. A. Freites, J. W. OConnor, D. J. Tobias, C. Mondragon-Ramirez, I. Vorobyov, A. D. MacKerell, and R. W. Pastor. Update of the charmm all-atom additive force field for lipids: Validation on six lipid types. *J. Phys. Chem. B*, 114(23):7830–7843, 2010.

[9] I. S. Joung and T. E. Cheatham III. Determination of alkali and halide monovalent ion parameters for use in explicitly solvated biomolecular simulations. *J. Phys. Chem. B.*, 112:9020–9041, 2008.

[10] K. Vanommeslaeghe, E. Hatcher, C. Acharya, S. Kundu, S. Zhong, J. Shim, E. Darian, O. Guvench, P. Lopes, I. Vorobyov, and A. D. Mackerell. Charmm general force field: A force field for drug-like molecules compatible with the charmm all-atom additive biological force fields. *J. Comp. Chem.*, 31(4):671–690, 2010.

[11] K. Vanommeslaeghe and A. D. MacKerell Jr. Automation of the CHARMM general force field (CGenFF) I: bond perception and atom typing. *J. Chem. Inf. Model.*, 52:3144–3154, 2012.

[12] K. Vanommeslaeghe, E. P. Raman, and A. D. MacKerell Jr. Automation of the CHARMM general force field (CGenFF) II: Assignment of bonded parameters and partial atomic charges. *J. Chem. Inf. Model.*, 52:3155–3168, 2012.

[13] M. J. Frisch et al. Gaussian 03, Revision C.02. Gaussian, Inc., Wallingford, CT, 2004.

[14] U. C. Singh and P. A. Kollman. An approach to computing electrostatic charges for molecules. *J. Comp. Chem.*, 5:129–145, 1984.

[15] B. H. Besler, K. M. Merz Jr., and P. A. Kollman. Atomic charges derived from semiempirical methods. *J. Comp. Chem.*, 11:431–439, 1990.

[16] W. Humphrey, A. Dalke, and K. Schulten. VMD – Visual Molecular Dynamics. *J. Mol. Graph.*, 14:33–38, 1996.

[17] GM Torrie and JP Valleau. Monte carlo free energy estimates using non-Boltzmann sampling: Application to the sub-critical lennard-jones fluid. *Chem. Phys. Lett.*, 28:578–581, 1974.

[18] S Kumar, D Bouzida, RH Swendsen, PA Kollman, and JM Rosenberg. The weighted histogram analysis method for free energy calculations on biomolecules.1. the method. *J. Comput. Chem.*, 13:1011–1021, 1992.

[19] B Roux. The calculation of potential of mean force using computer simulations. *Comput. Phys. Commun.*, 91:275–282, 1995.

[20] Alan Grossfield. Wham: the weighted histogram analysis method. *http://membrane.urmc.rochester.edu/content/wham.*

[21] Woo HJ and Roux B. Calculation of absolute proein-ligand binding constants with the molecular dynamics free energy perturbation method. *Methods Mol. Biol.*, 443:109–120, 2008.

[22] Dan Gordon, Rong Chen, and Shin-Ho Chung. Computational methods of studying the binding of toxins from venomous animals to biological ion channels: theory and applications. *Physiological reviews*, 93(2):767–802, 2013.

[23] FR Beierlein, GG Kneale, and T Clark. Predicting the effects of basepair mutations in DNA-protein complexes by thermodynamic integration. *Biophys. J.*, 101(5):1130–1138, 2011.

[24] R. W. Zwanzig. High temperature equation of state by a perturbation method. I. Nonpolar gases. *J. Chem. Phys.*, 22(8):1420–1426, 1954.

[25] S Lee, J.M.J. Swanson, and G.A. Voth. Multiscale simulations reveal key aspects of the proton transport mechanism in the clc-ec1 antiporter. *Biophys. J.*, 110:1334–1345, 2016.

[26] S Lee, H.B. Mayes, J.M.J. Swanson, and G.A. Voth. The origin of coupled chloride and proton transport in a cl/h+ antiporter. *J. Am. Chem. Soc.*, 138:14923–14930, 2016.
